# Supplementary material for: Are carbon emissions trading and green financial instruments synergistic? -Comprehensive quantitative research based on content analysis
Source: PLoS One. 2024 Mar 7;19(3):e0298601. doi: 10.1371/journal.pone.0298601 (PMC10919601; doi:10.1371/journal.pone.0298601)
Supplement: S1 Data — (ZIP) [file pone.0298601.s001.zip › Data/地级市Z_F工作报告.zip!/╣≤╓▌/╣≤╤⌠╩╨/└┤╫╘╣1⁄2╓┌║┼í╛┬φ┐╦╩2╛▌═°í┐_2006.docx]

各位代表：

根据《中共贵阳市委关于制定贵阳市国民经济和社会发展第十一个五年规划的建议》，市人民政府制定了《贵阳市国民经济和社会发展第十一个五年规划纲要（草案）》。现在，我代表市人民政府向大会作报告，请各位代表连同《纲要（草案）》一并审议，并请各位政协委员和其它列席人员提出意见。

“十五”时期国民经济和社会发展的回顾

过去五年，在省委、省政府以及市委的领导下，在市人大和市政协的监督、支持下，我们坚持以邓小平理论和“三个代表”重要思想为指导，牢固树立和落实科学发展观，着力构建社会主义和谐社会，紧紧围绕建设大贵阳和生态经济市的目标，大力实施“工业强市、环境立市、科教兴市、人才强市和可持续发展”战略，团结和依靠全市各族人民，抢抓机遇，深化改革，扩大开放，加快发展，全面完成了市十届人大第五次会议通过的“十五”计划的各项目标任务。“十五”计划的五年，是贵阳市在西部大开发中攻坚克难、打好发展基础阔步前进的五年，是经济社会发展又快又好、城乡面貌变化最大、人民群众得到实惠最多的五年，五年的奋进铸就了贵阳市经济和社会发展令人瞩目的辉煌成就。

一、国民经济持续快速增长，综合经济实力大幅度跃升

“十五”期间，全市国民经济保持了持续快速增长势头，综合经济实力迈上新台阶。全市生产总值从2000年的269.12亿元，增加到2005年的525.62亿元，年均增长12.8%，比2000年翻了近一番，其中，三次产业年均分别增长5.9%、14.2%和12.2%。财政总收入和地方财政收入分别由2000年的61.41亿元和24.15亿元，增加到2005年的133.15亿元和62.50亿元，年均分别增长16.74%和20.95%，各区（市、县）地方财政收入均突破1亿元，7个区（市）县进入全省经济强县前十强。城市居民人均可支配收入达到9928元，农民人均纯收入达到3135元，分别比2000年增加3475元和1031元，年均分别增长8.5%和6.6%。金融机构存贷款余额分别达1260.43亿元和1038.18亿元，是“九五”期末的2.4倍和2.6倍。

二、经济结构调整取得突破性进展，质量和效益显著提高

全市经济发展紧紧围绕结构调整这一主线，采取有力措施，推进经济结构的不断优化升级。三次产业占生产总值的比重由2000年的9.0：48.8：42.2调整为2005年的6.6：47.5：45.9。非公有制经济发展迅速，非公有制经济比重从“九五”期末的32.2%提高到2005年的41.6%，成为拉动我市经济发展的重要力量。工业结构调整取得重大突破，全市高技术产品增加值完成35.35亿元，为2000年的2.5倍。磷化工、铝及铝加工、煤化工、装备制造、卷烟、能源等现实优势产业对全市经济的支撑作用进一步增强，以电子信息产品、新材料、现代中药业为代表的一批高新技术产业快速发展，循环经济试点项目全面启动。农业产业结构调整步伐加快，特色农业及名牌产品不断涌现，农业产业化龙头企业不断发展壮大，农业继续向优化种植业和养殖业的方向推进。第三产业发展领域不断扩大，整体水平继续提升，商品流通发展迅速，市场保持繁荣兴旺，交通、通信、旅游、信息、社会服务等发展尤为迅速，第三产业对国民经济的贡献明显提高。生产力布局和结构进一步优化，县域经济实力不断增强，2005年城镇化率提升到63.4 %。

三、固定资产投资持续增长，城乡面貌发生巨大变化

全市固定资产投资大幅增长，成为推动经济增长的主导力量。累计完成全社会固定资产投资1221.94亿元，为“九五”时期的3倍。以交通为重点的基础设施建设取得突破性进展，2003年在全省率先实现乡乡通油路，五年累计改建、改造县、乡道路2400公里，村级路2358公里，“十五”时期交通基础设施建设投入是前50年投入总和的60倍。大力实施退耕还林、“二环林带”等生态建设工程，荣获首个“国家森林城市”和“全国绿化模范城市”称号。按照“水、电、路、气、房、林、电视、电话”十字方针，加大农村基础设施建设，极大地改善了农民生产生活条件。实施了“渴望工程”、“解困工程”、“万户小水池工程”、病险水库治理工程，新建、改建了一批水利骨干工程，解决38万人的饮水困难，基本解决全市农村群众饮水困难问题；完成农村茅草房改造工程，使15万农民告别世代居住的茅草房；1171个行政村全部实现“模改数”，5040个自然村寨通了广播电视。大力实施“三轮驱动”战略，全力推进新区建设、旧城改造、小城镇建设，城市综合服务功能日益增强，城乡面貌发生巨大变化，城市化进程不断向前迈进。2005年，城市人均道路面积达6.3平方米，城市绿化覆盖率达41.3%，人均公共绿地面积达9.27平方米，城市污水处理率达22%，城市燃气气化率达97%。城市基础设施进一步改善。实施“南明河三年变清”系列工程，实现“水变清、岸变绿、景变美”的目标，获得了中国人居环境范例奖。金阳新区开发建设取得突破性进展，提前5年完成金阳新区17平方公里范围内基础设施建设，建成金阳新区道路网主骨架，完成市级行政中心、学校、医院、公园、房地产等一批城市功能配套设施，金阳新区已成为全市乃至全省高新技术产业聚集的平台。城市管理水平不断提高。以创建全国、全省文明城市为主线，以建设宜居城市为目标，着力打造“林城”品牌，注重城市“绿化、美化、亮化、稀化、净化”，大力实施“绿色”、“碧水”、“蓝天”、“宁静”四大工程，保护和改善城市生态环境，进一步提升了城市形象和品位。

四、体制改革取得重大进展，对外开放取得丰硕成果

国有企业改革向纵深推进，成功实施一批国有企业的资产重组，完成一批国有资本从一般竞争性领域中退出的任务，国有大中型企业历史包袱明显减轻，国有资产监管体制初步建立。农村税费改革试点取得显著成效，启动农村综合改革试点，全面免除农业税，完成农村信用社产权制度改革；全面放开粮食购销市场，粮食流通体制发生根本性的转变。行政审批制度改革取得阶段性成果，政府职能进一步转变。以部门预算管理、财政国库管理和政府采购制度为重点的财政体制改革取得新的成绩，财政转移支付制度进一步完善。金融改革稳步推进，地方中小金融机构和民营金融机构加快发展。投资主体多元化的格局基本形成，企业投资主体地位进一步巩固，政府投资行为进一步规范。对外贸易迈上新台阶，累计完成进出口贸易总额42.6亿美元，年均增长18.3%，比“九五”时期增长近一倍。招商引资成效显著，积极参与泛珠三角、长三角、环渤海三大经济圈和海峡西岸经济圈及香港、澳门等区域合作，“十五”期间，全市共批准外商投资企业140家，合同利用外资2.7亿美元，实际直接利用外资2.3亿美元，沃尔玛、京瓷、西洋肥业、山东兖矿等20多个国际国内的著名企业落户贵阳。

五、社会各项事业全面进步，构建和谐社会开创新局面

大力推进“科教兴市、人才强市”战略的实施，不断加大对社会薄弱环节的投入，社会各项事业全面进步，经济社会协调发展，构建社会主义和谐社会成效明显。累计投入科技三项费2.34亿元，是“九五”时期的2.82倍，科技体制改革进一步深化，市属科研机构体制改革取得重大进展，科技创新能力增强。人才队伍建设力度进一步加大，人才数量持续增长，人才素质不断提升。教育事业发展迅速，教育改革全面推进，教学质量稳步提高，累计投入国家财政性教育资金50.29亿元，年均增长15.3%，完成贵阳学院组建，贵阳一中新校、实验三中建成并投入使用。“两基”成果得到进一步巩固和提高，累计投入9000万元对全市农村中小学校危房进行改造，建成新校舍23万多平方米，农村中小学校办学条件明显改善。卫生事业改革与发展取得长足进步，疾病预防控制体系、医疗救助体系、卫生监督体系和农村卫生服务体系建设加强，开展城市医疗救助试点和新型农村合作医疗制度试点，全市婴儿死亡率控制在12‰以内，孕产妇死亡率大幅下降。突发公共卫生事件应急处理能力进一步提高，成功防止非典疫情进入我市。人口与计划生育工作力度加大，人口自然增长率由2000年的8.8‰降低到5.71‰。文化产业蓬勃发展，文化体制改革深入推进，以县级“两馆”为重点的基层文化设施建设得到加强。精神文明建设和民主法制建设取得新的进展，依法行政能力进一步提高。就业形势基本稳定，累计实现城乡统筹就业人员28.17万人，其中：城镇失业人员就业12.53万人；下岗失业人员再就业6.81万人；组织农村剩余劳动力转移8.83万人，城镇登记失业率控制在4.2%以内。建立了基本养老、基本医疗、失业、工伤、生育等五险齐全的社会保险制度，“两个确保”100%的成果进一步巩固，“三条保障线”各项政策全面落实，城市低保实现“应保尽保”，农村低保全面铺开。城乡居民收入水平不断提高，扶贫攻坚取得了阶段性成果，居民家庭用于教育、文化、保健和旅游等方面的支出增加。社区建设稳步推进；社会治安综合治理成效显著；安全生产形势总体趋于平稳；和谐贵阳建设取得重大进展。公安、司法、监察、新闻、出版、体育、审计、统计、物价、工商、质监、人事、民政、民族、宗教、人防、社会科学、知识产权、外事侨务、对台、市志、档案等各项事业取得明显进步。

2005年，贵阳市的主要经济指标实现了快速增长，取得了较大突破。全年生产总值达到525.62亿元，比上年增长14.6%，这是自1986年以来20年期间增幅最高的一年。固定资产投资完成343.54亿元，比上年净增50亿元。财政总收入完成133.15亿元，增长20.2%，地方财政收入完成62.50亿元，增长25.7%。实现社会消费品零售总额201.84亿元，增长15%。实际直接利用外资6045万美元，引进市外资金161.01亿元，分别增长15.4%、31.5%。2005年我市经济社会发展取得了较好的成绩，质量、效益跃上新台阶，为完成“十五”计划画上了圆满的句号，也为顺利实施“十一五”规划奠定了坚实的基础。

各位代表，“十五”期间我市经济社会发展取得的成绩，是省委、省政府以及市委正确领导和市人大、市政协有效监督的结果；是各级各部门大力支持的结果；是全市各族干部群众抢抓机遇、加快发展、奋力拼搏、团结奋斗的结果。在此，我代表市人民政府，向全市工人、农民、知识分子、干部，向各民主党派、工商联、无党派人士、人民团体，向驻筑人民解放军指战员、武警部队官兵、公安干警，向所有关心和支持我市改革开放和现代化建设的各界人士，表示衷心的感谢并致以崇高的敬意！

在充分肯定成绩的同时，我们也清醒地认识到在前进道路上还面临不少困难和问题：经济结构不合理，自主创新能力不强，粗放型增长方式尚未根本转变，经济社会发展与人口、资源、环境的矛盾仍较突出，解决“三农”问题的任务仍很艰巨，城乡就业和社会保障仍然面临很大压力，制约发展的体制机制障碍仍然存在，社会利益关系的处理难度较大。对于这些问题，我们将高度重视，坚持用发展的思路和改革的办法妥善加以解决。

“十一五”时期的奋斗目标和主要任务

“十一五”时期是我市加快建设小康社会、率先实现历史性跨越的关键时期，是我市发展承前启后、实现重点突破的关键五年。根据《中共贵阳市委关于制定第十一个五年规划的建议》，“十一五”时期我市经济社会发展的总体要求是：以邓小平理论和“三个代表”重要思想为指导，坚持科学发展，着力自主创新，完善体制机制，促进社会和谐，牢牢把握全面贯彻落实以科学发展观统领发展全局的六条原则，立足新起点，增创新优势，实现新跨越，深入实施工业强市、科教兴市、人才强市、环境立市和开放带动战略，调整经济结构，转变经济增长方式，推进经济发展转型，壮大特色优势产业，发展循环经济，大力推进社会主义新农村建设，加快新型工业化、农业产业化、农村城镇化和城市现代化步伐，把加快发展与全面协调发展结合起来，促进节约发展、清洁发展、安全发展和可持续发展，力争经济总量、综合实力、生态环境、居民生活水平再上一个大台阶，开创经济建设、政治建设、文化建设、和谐社会建设的新局面，“做表率，走前列”，努力率先在全省实现经济社会发展的历史性跨越。

“十一五”期间经济社会发展的主要目标是：到2010年，全市生产总值突破1000亿元，人均生产总值突破3000美元，为全面建设小康社会、基本实现现代化奠定坚实基础；资源综合利用水平有新提高，单位地方生产总值能源消耗比“十五”期末降低20%，生态环境明显改善；结构调整取得新突破，特色优势产业发展壮大，形成一批拥有自主知识产权和知名品牌、竞争力较强的大型企业、企业集团；建立起较为发达的交通运输体系和较为完备的城乡公共服务体系；社会主义市场经济体制比较完善，开放型经济达到新水平；科技、教育、文化、卫生等社会事业全面发展，城乡居民生活质量和水平明显提高；民主法制建设和精神文明建设进一步加强，和谐社会建设取得新成效。

“十一五”期间我市经济社会发展的主要预期调控指标是：生产总值年均增长13%以上；人均生产总值年均增长12%左右；全社会固定资产投资年均增长16%左右，累计比“十五”翻一番以上；地方财政收入年均增长13%左右；社会消费品零售总额年均增长14%左右，累计增加城镇就业20万人以上，城镇登记失业率控制在4.5%以内；城镇化水平提高到68%左右；城市居民人均可支配收入和农民人均纯收入年均增长8%左右；人口自然增长率控制6‰以内。

围绕上述总体要求和目标，我们要全力以赴做好以下工作：

一、以增强城市经济整体竞争力为目标，推进产业结构优化升级和增长方式转变，增创产业发展新优势

（一）加快推进新型工业化，增强工业经济的聚集力和竞争力，努力实现节约发展、清洁发展、安全发展和可持续发展。切实推进工业经济发展转型，推进经济发展由“三高一低”（高能耗、高成本、高污染、低效益）的传统模式向“三低一高”（低能耗、低成本、低污染、高效益）的现代模式转变，是我市工业经济实现历史性跨越的根本途径，也是我市适应现代经济发展的必然选择。必须立足资源、能源和产业基础优势，坚持走新型工业化的道路，着力发展高新技术产业，改造提升传统产业，大力发展循环经济，招商引资聚集产业，自主创新提升产业，依托开发区发展产业，培育壮大特色优势产业，提高产业集中度和精深加工水平。整合区域科研资源，加强与省内外知名科研机构、高等院校、军工企业的合作，充分发挥企业在科技创新中的主体作用，在努力提高原始创新能力的同时，积极发展集成创新和引进消化吸收再创新，大力发展先进生产能力，促进产业优化升级。进一步加快三个开发区的发展，认真落实支持开发区发展的政策措施，加大基础设施建设力度，加快园区高新技术企业的引进、投产、达产，形成高新产业集群，实现开发区的跨越式发展。培育一批具有自主知识产权、核心竞争力强、市场占有率高的强势企业、名牌产品和优势产业集群，确保全市工业增加值年均增长15%以上，形成6个年销售额超过百亿元以上的优势产业集群，8个年销售额超过50亿元的大型企业或企业集团，培育10个国家级名牌产品。

大力发展电子信息产品、新材料、现代药业等高新技术产业。抓好国家新型电子元器件产业基地、微硬盘、数字平板电视、应用软件开发和集成等重大项目建设，发展上、下游配套产品。建设好国家级新材料产业化基地，重点发展能源、环保、电子、化工、建材、机械等新材料，加快金属及聚合物改性材料、纳米改性材料等的产业化，支持“高解像超微柱型”立体感光材料、通讯终端产品、铜箔产业化等项目建设。

大力发展铝工业、磷化工、煤化工等现实优势产业。按照循环经济要求，走铝、磷、煤、电联营和集群化发展的道路，努力把我市打造成为全国重要的新型铝工业和磷化工基地。重点发展氧化铝和铝的深加工产品，完成中铝贵州分公司氧化铝、电解铝扩建项目建设，争取新建一个年产80—120万吨氧化铝及深加工项目。抓好矿肥结合、矿化结合，重点建设中化开磷（集团）、西洋肥业（集团）、兖矿开磷合成氨磷铵项目和一批黄磷深加工、黄磷尾气、磷渣综合利用项目。依托中国化工集团，规划建设大型煤化工项目，重点建设贵州水晶集团甲醇、羰基合成醋酸、甲醛等项目。

加快发展中药、烟草、特色食品等特色产业。以发展具有自主知识产权的现代医药产品为重点，加强中药、苗药新产品开发。加强中药材GAP基地建设，抓好以修文扎佐、清镇、乌当、花溪医药工业园为重点的一批医药工业园建设，建设符合GSP要求的以经营贵州地道中药材为主的大型中药材专业批发市场，培育益佰制药、同济堂等一批强势企业和知名品牌，提高产业集中度、知名度和竞争力。支持黄果树烟草集团完成贵阳卷烟厂搬迁改造，形成200万大箱年生产能力。抓好食品工业园区建设，依托老干妈风味食品有限公司等一批龙头企业，加快发展以辣椒、乳制品和调味品为重点的特色食品工业。

振兴装备制造及汽车零部件等先进制造业。抓住国际装配制造业向中国转移的有利时机，推动军工、民用结合，加快产业升级。重点建设矿山机械厂年产7.4万台液压挖掘机、贵州轮胎公司年产500万条半钢子午线轮胎、永青示波器厂年产1万套工程机械电子监测及节能控制系统等项目。

（二）加快发展以旅游业为龙头的现代服务业，促进第三产业优化升级，增强省会城市的服务功能。坚持市场化、产业化、社会化和国际化方向，加快实施重点带动和品牌发展战略，把青岩古镇历史文化旅游区、红枫湖—百花湖亚高原生态休闲旅游区、乌江峡红色旅游景区、南江峡谷喀斯特生态旅游区等建设成为全省乃至西部的重要旅游目的地。进一步发挥全省旅游重要目的地和服务中心的优势，围绕“行游住食购娱”，加强旅游交通、宾馆、信息咨询服务中心、标识系统、购物点等配套设施的建设，推进旅游设施建设和管理市场化，加大旅游产品推介力度，加快引进和培育一批优强旅游企业，提高旅游业发展水平和整体竞争力。旅游总收入年均增长20%以上。

加快发展现代物流业。推进物流基础设施和物流园区建设，发展一批社会化、专业化的大型物流企业，支持企业联合开展现代物流服务。加快建设二戈寨—改貌、龙洞堡航空港、扎佐—阳关等物流中心，努力把贵阳建设成为全省乃至西南地区重要的物流中心。物流业营业收入年均增长20%以上。

积极发展信息、中介、咨询、金融等现代服务业；改造提升商贸流通、餐饮等传统服务业；发展连锁经营、集中配送、电子商务等新型服务业；积极发展以居民住宅为重点的房地产和装饰装修业；拓展文化、社区服务等需求潜力大的服务业。建立公开、透明、管理规范的准入制度，强化对服务业发展的政策支持和引导。服务业增加值年均增长13%左右。

二、以建设社会主义新农村为主线，积极统筹城乡发展，增创“三农”工作新优势

（一）继续加强农村基础设施建设，进一步改善农村生产生活条件。坚持工业反哺农业、城市支持农村和“多予少取放活”的方针，切实改善广大农村的生产生活条件和整体面貌，继续抓好以“水、电、路、气、房、林、电视、电话”为重点的农村基础设施建设。积极配合黔中水利枢纽工程建设，加快以渔洞峡、席关水库等为重点的一批中小型水利工程建设，逐步解决农村饮水安全问题，使乡村自来水普及率达75%以上。完善水利灌溉配套设施，新增有效灌溉面积3万亩。加强基本农田建设，搞好中低产田土改造。全面完成农村电网改造，实现城乡用电同网同价。加快农村等级公路和村寨串户路建设，推进农村公路养护市场化。加快农村沼气池建设，3年内全面完成农村18.5万口沼气池建设。加快农村危房改造。加强农村广播电视网络、通讯网络和互联网络建设，发展农村远程教育，实现5户以上自然村通广播电视率达95%，完善市、县、乡三级信息网络建设。

（二）加大农业产业结构调整力度，千方百计增加农民收入。按照发展都市农业、生态农业、高效农业、特色农业的定位和要求，大力发展农业产业化经营，扶持龙头企业和农村合作组织的发展，加快农产品质量标准体系和动植物检疫体系建设，规划建设一批特色优质农产品重点发展区、农业标准化生产基地、科技示范基地、观光园区和生态养殖小区，打造农业知名品牌，提高农业综合经济效益，确保第一产业增加值年均增长5.5%。加快中药材、辣椒、粮油、畜产品、果品等农产品加工型企业的发展，建设无公害优质农产品果树、蔬菜、中药材等八大优势产业带，加强反季节蔬菜、花卉苗木等养殖基地和经果林基地建设。大力发展乡镇企业和城镇非公有制经济，加强农业技术服务，加大农村劳动力培训力度，积极组织农村富余劳动力外出务工，切实增加农民收入。到2010年，从事非农产业劳动力人数达到农村劳动力总数的40%。

（三）切实加大对农村的投入力度，积极发展农村公共事业。落实新增教育、文化、卫生、计划生育等事业经费主要用于农村的制度，力争使农村社会事业发展得到显著改善。巩固提高农村“普九”成果，实施“农村中小学现代远程教育工程”和“户均一名高中生、村均一名大学生”工程，大力发展农村职业教育，实现农村义务教育全部免费，培育社会主义新型农民。加强农村公共卫生和基本医疗服务体系建设，完成乡镇卫生院的改造及基本设备配套，基本建立新型农村合作医疗制度，使农村初级卫生保健水平达到小康型合格县标准。加强农村文化建设，倡导健康、文明、科学的生活方式，不断提高农村社会文明程度。建立健全农村法律服务体系，加强农村普法教育，增强农民法制观念。

（四）加大新阶段扶贫开发力度，全面解决农村贫困人口的温饱问题。按照整村推进、产业化扶贫和劳动力转移培训的要求，全面推进贫困地区的经济社会发展。对贫困人口进行分类指导，采取开发式扶贫、易地扶贫搬迁、定期救助等方式，使农村贫困人口实现脱贫或基本生活有保障。继续加强对口帮扶、社会力量帮扶和定点扶贫，开展项目扶贫、产业化扶贫、教育扶贫、卫生扶贫、科技扶贫，逐步建立抑制返贫的长效机制。

三、以促进区域经济协调发展为着力点，着力优化生产力空间布局，增创中心城市发展新优势

（一）优化生产力布局。以将贵阳市建成西南地区重要的区域经济中心和黔中产业带的龙头为目标，强化产业特色，提高产业集聚程度，努力形成“二四四六”的产业空间布局。强化中心城区商贸、金融、信息、科技、旅游服务的区域中心地位，三个开发区大力实施以增强技术创新能力为核心的“二次创业”战略，重点发展高新技术产业，使其成为高新技术研究开发的核心基地。一市三县重点发展资源型的优势产业，形成优势互补、竞相发展的特色经济体系。同时，以全省第二轮经济强县建设为载体，下放事权，增强财力，提高县域经济统筹能力，力争“十一五”期末各区、市、县生产总值比2005年翻一番并全部进入全省经济强县行列。

（二）推进“贵阳城市经济圈”建设。按照“市场主导、政府推动、互惠互利、从实起步”的原则，加强与经济圈内各区域的沟通与合作，打破行政区划局限和体制障碍，创新合作机制，推进区域市场一体化。充分发挥交通枢纽和通信网络的作用，促进区域便捷交通网络形成和信息资源共享，力争建成具有较强国际竞争力的新型磷产业、铝产业及国内重要的先进制造业、高新技术产业、烟草、现代中药及特色食品、旅游基地。

四、以交通运输、能源水利、公用设施、信息等为重点，着力优化和完善城乡基础设施体系，增创城市服务功能新优势

（一）加强交通运输体系建设。对外交通与省“三纵三横八联八支”骨架公路体系建设相衔接，市内交通以“一纵两横三环线”、“一环一横七射线”城乡公路网体系建设为支撑，推进城市及县、乡、村公路建设和改造，打造城区半小时、市域一小时交通网络，基本形成网络通畅、性能良好的道路网络。重点实施贵阳过境段高速公路、西出口高速公路、扎南高速公路、贵开路、桐木岭至惠水公路、清镇至金阳高等级公路等项目，力争等级公路建设达2000公里以上。铁路以强化枢纽节点为重点，重点建设贵阳铁路枢纽工程、境内川黔线复线、黔桂线扩能改造等工程，提高通达深度和快捷程度。积极配合龙洞堡机场扩建及配套设施的建设。

（二）加强能源水利建设。加快电源点建设，支持清镇电厂异地搬迁改造，积极发展中小水电站。加强城乡电网建设和改造，提高供电能力。加强煤炭资源勘探和煤矿建设，重点发展年产30万吨以上煤矿。加强以解决工程性缺水为重点的水利建设，加快一批中小型水利工程建设，加强病险水库改造，完善缺水区域生产、生活用水和农田灌溉配套设施。积极开展空中水资源开发试点工作。继续实施红枫湖、百花湖、阿哈水库等城市饮用水源水库的综合治理工程，构建城乡一体的水利网络，提高城市防洪减灾能力。

（三）加强城市公用设施建设。改造老城区供水管网，新建金华水厂、松柏山水厂，提高城镇供水普及率，使饮用水水质达到国家规定标准。加快建设和管理使用好污水处理厂，建设和改造城镇排水及污水收集系统，推广生活污水无害化处理技术。加强煤气气源点和储备站建设，完善网管建设。实施城市综合管网入地工程。建立以公共交通为主、多种交通方式互补的现代城市交通系统。实施城市客运车辆站场建设项目，加快公交枢纽站、客运站、停车场建设。推进城市无障碍公共设施建设。加快再生资源交易中心、南郊垃圾填埋场及全省危险废弃物处置中心等项目建设，推进城市垃圾的综合利用。

（四）加强信息基础设施建设，构建“数字贵阳”。积极推进贵阳地理信息系统建设，加快发展第二代互联网和用户驻地宽带接入网，推进宽带通信网、数字电视网和互联网“三网融合”及“金”字工程建设，加强重点行业和重点领域信息技术应用。统筹城乡信息化，进一步提高农村电话普及率和广播电视人口综合覆盖率。

五、以建设最适宜创业发展和生活居住城市为发展方向，努力提高城市建设和管理的现代化水平，增创推进城镇化新优势

（一）强力推进金阳新区建设。坚持高起点规划、高标准建设、高强度投入、高效能管理，深入推进金阳新区开发建设，把新区17平方公里建设成为功能完善、产业发展、品位较高的现代化新城区，完成40平方公里的基础设施建设。力争完成贵州大学搬迁工程，加快大学城、体育中心、会展中心、金阳医院、科技馆、图书音像城、广电中心、客货运物流设施、污水处理厂等一批功能性基础设施及社会发展项目建设。进一步创新体制机制，加大招商力度，引进一批高新技术企业和有知名度、有实力的房地产开发企业，加快产业培育和人口聚集，逐步把金阳新区建设成为生态型、数字化、园林式城市，成为我市经济社会发展的重要增长极和贵州省的“浦东”。

（二）加快老城区成片改造，提高城市管理水平。以规划为龙头，落实“稀化”要求，进一步完善老城区城市功能，力争完成“城中村”改造，加大老城区成片改造建设力度。重点建设一批枢纽型、功能性、具有较强集散能力的城市基础设施，完善市区交通网络和市区对外交通体系，优化城市公交线网布局。加强停车场（库）的规划、建设和管理，严格车辆停放管理，提高中心城区道路通行能力。加快实施“贸改超”工程，切实解决占道经营、马路市场等问题。继续巩固和扩大南明河三年变清的成果，着力抓好截污沟、污水处理厂的建设，实施市内主要出口公路、居民住宅小区全面绿化，推进山体公园建设，科学绿化、美化、亮化、净化、稀化城市环境，促进人与环境的和谐发展。进一步完善城市管理体制，建立市、区（县）、街道（镇）相互衔接、合理分工、规范高效的城建、城管综合执法体制。

（三）有序发展小城镇。加快实施新一轮城镇规划。创新城镇建设和管理体制，完善户籍和流动人口管理办法，积极引导产业和人口向城镇聚集和转移，适当超前加快城镇基础设施建设，提高城镇建设和管理水平。切实抓好国家、省、市重点小城镇和示范小城镇的建设，逐步形成梯次结构合理、区域分工明确、基础设施配套、社会文明进步、城乡协调发展、生态环境优美且各具特色的城镇体系。

六、以循环经济和环境保护为抓手，努力加快资源节约型、环境友好型社会建设步伐，增创生态环境新优势

（一）大力发展循环经济，建设节约型社会。切实抓好循环经济试点城市的各项工作，建立循环经济建设绩效考核评估体系和激励、约束机制，推进节能节水节地节材与资源综合利用和清洁发展，逐步形成节约型增长方式和消费模式。以磷化工、铝加工和煤化工等高耗能行业为突破口，加快循环经济生态工业试点建设，抓好示范项目建设，建设好开阳磷煤化工、清镇煤化工、息烽磷复合肥等生态工业示范基地。认真落实国家税收优惠政策，制定促进发展循环经济的财政、价格和收费政策，基本建立企业自觉实施清洁生产的机制和管理体制，逐步形成以资源节约型、清洁生产型、生态环保型为明显特征的新型工业化格局。以农业科技示范园区和生态经济示范村为重点，推广生态农业模式。加强宣传教育，强化节约意识，引导形成健康文明、节约资源的消费模式，创建一批节约型机关、节约型企业、节约型社区。

（二）加强生态建设与环境保护，建设生态经济市。以争创国家环保模范城市和国家人居环境奖为目标，深入实施“绿色”、“碧水”、“蓝天”、“宁静”四大工程，扎实推进生态经济市建设。加强自然保护区和生态示范区的建设和管理，继续实施石漠化治理、退耕还林还草、天然林保护、长江水源保护、喀斯特生态系统保护和环城、环湖林带等生态工程建设，促进自然生态恢复，提高森林覆盖率。整顿矿业秩序，加强地质灾害防治工作。进一步加强全市水资源的统一规划、利用、保护和管理，将现有高污染、高能耗的工业企业从水源保护区逐步迁出，切实保护红枫湖和百花湖等饮用水水源。继续抓好环境保护和治理，加强工业大气污染和汽车尾气、扬尘、饮食油烟等污染的防治，妥善处理生活垃圾和危险废物，逐步推行垃圾分类处理，加强城市环境噪声的综合整治。进一步健全环境监管体制，加大环保执法力度，实施排放总量控制、排放许可和环境影响评价制度。大力发展环保产业，建立社会化多元化环保投融资机制，推进污染治理市场化进程。

七、以深化改革、扩大开放为动力，进一步完善社会主义市场经济运行机制，增创投资环境新优势

（一）深化国有企业改革，引导和促进非公有制经济加快发展。加快国有经济布局和结构战略性调整步伐，促进国有资本的优化配置。深化国有资产监督管理体制改革，建立健全出资人制度和国有资产授权经营管理制度，落实国有资产经营责任制，加强国有资产管理和监督。加快国有大型企业股份制改革，加快建立现代企业制度，完善公司法人治理结构。深化集体企业改革，发展多种形式的集体经济。鼓励和支持非公有制经济参与国有企业改革，进入金融服务、公用事业、基础设施等领域。充分发挥工商联、商会、行业协会等组织的作用，引导、促进非公有制企业加强管理，使非公有制经济在国民经济中的比重提升到50%以上。

（二）深化财税体制和投融资体制改革。加快公共财政体系建设，逐步建立规范的公共财政框架和财政转移支付制度，进一步提高社会公共服务和社会保障支出的比重。完善市、区（市、县）财税分配体制。继续深化部门预算、国库集中收付、政府采购和收支两条线管理制度改革。深化投资体制改革，完善投资核准和备案制度，健全政府投资决策责任制度，建立市场引导投资、企业自主决策、银行独立审贷、融资方式多样、中介服务规范、宏观调控有效的新型投资体制。积极做好项目前期工作，推行项目法人责任制、招投标制、工程监理制、合同管理制，建立投资风险约束机制。争取引进外资金融机构到我市设立分支机构。

（三）深化农村各项改革。巩固农村税费改革成果，全面推进农村综合改革，基本完成乡镇机构、农村义务教育和县乡财政管理体制等改革任务。落实最严格的耕地保护制度，加快征地制度改革，完善征地程序和补偿机制，探索被征地农民的社会养老保险路子，解决好被征地农民的长远生计问题。加快农村合作经营制度改革，实现土地适度规模经营，引导社会资本进入现代农业开发领域。改革和创新农村金融体系，加大对农户和龙头企业的金融支持力度。

（四）推进现代市场体系建设。进一步开放、培育和完善市场，形成统一、开放、竞争、有序、功能健全、符合市场经济运行规则的市场体系。继续培育土地、技术和劳动力等要素市场，积极发展各类中介组织，完善商品和要素价格形成机制。积极开拓农村消费市场，健全农产品流通体系和购销服务体系。加强市场监管，加快建设社会信用体系，建立失信惩戒制度，维护和健全市场秩序，建设“诚信贵阳”。

（五）扩大对内对外开放，提高招商引资质量。始终把治理、巩固、提升、创优发展环境作为加快发展的基础工程和各级政府的重要职责，完善投资环境监测、考核、评价制度，营造良好的法制环境、信用环境和服务环境。抓住国际国内产业加速转移的机遇，紧紧围绕我市优势产业招商，继续把珠江三角洲、长江三角洲、环渤海及海峡西岸经济圈、港澳地区作为招商重点，加大对东盟、日本、韩国及欧美的招商力度，力争引进世界及国内500强企业20家。注重引进先进技术、管理经验和高素质人才，做好引进技术的消化吸收和创新提高。坚持“引进来”和“走出去”相结合，鼓励和支持有条件的企业参与国际国内竞争。加强贸易合作，大力发展开放型经济。转变对外贸易增长方式，优化出口产品结构，创造条件建立出口加工区，切实提高对外贸易的质量和效益。加强与东部地区和泛珠三角经济区、成渝经济区各方合作，抓住建立中国东盟自由贸易区的机遇，发展多种形式的对内对外经贸关系，推动区域经济合作。全市外贸进出口总额年均增长10%以上。

八、以实施科教兴市、人才强市战略为着力点，努力建设创新型城市，增创综合创新能力的新优势

（一）发展各类教育，提高全民教育水平。继续深化教育体制改革，在全社会形成推进素质教育的良好环境，促进各级各类教育协调发展，建设学习型社会。巩固提高“普九”成果，统筹城乡基础教育发展，城乡义务教育实现标准化办学。继续增加各级财政对义务教育的投入，加强教育基础设施建设，加强师资队伍建设，切实提高师资特别是职教和农村师资水平。完成县级示范幼儿园和中心幼儿园建设。支持发展民办教育。率先在全省基本普及高中阶段教育。加快发展职业技术教育及职业技术培训，组建贵阳职业技术学院、贵阳护理职业学院和贵阳市职业教育中心，扶持县级职教中心建设。基本实现高中阶段在校生中普通高中与中等职业教育比例大体相等。加快发展高等教育，建好、办好贵阳学院。抓好现代远程教育。到2010年，全市新增劳动力平均受教育年限达到10年。

（二）积极推进科技进步和创新。实施科技创新工程，推动循环经济技术创新体系建设，强化企业技术创新的主体地位和企业家的核心作用，提高企业技术创新能力。加强高新技术的研究开发，建设中国西部（贵阳）高新技术产业研发生产基地。深化科技体制改革，优化创新环境，整合科技资源，初步建立“结构优化、布局合理、精干高效、纵深配置”的科技创新体系与运行机制。争创国家可持续发展实验区。加强知识产权保护，广泛开展科普工作。繁荣发展哲学社会科学，加强应用研究，发挥其对我市经济社会发展的重要作用。

（三）加强人才资源开发和利用。大力实施人才强市战略，继续实施“高层次党政人才培养、企业家培养、高技能人才培养、农村实用人才培养”等四大工程，大力培养各类人才。完善优惠政策，积极培养和引进我市重要产业、重点学科、重大项目急需的高层次人才和紧缺人才。加快人才评价向个性化评价和社会化评价机制转变，建立和完善人才评价体系和激励机制。加大人力资源开发的投入，推进市场配置人才资源，营造人才辈出、人尽其才的社会氛围。

（四）积极发展文化事业和文化产业。深化文化体制改革，逐步完善文化管理体制和文化产品生产经营机制。进一步发展文学艺术、新闻出版、广播影视、档案、市志等文化事业，稳步推进文化事业单位的改革。大力发展文化产业，培养和造就一批优秀文化人才，打造一批优秀作品，支持和培育在全国有影响的名牌专业团体。组建贵阳日报报业集团，支持实施“大地之舞”计划，打造白云风筝节、阳明文化节等地方品牌。加快文化产品市场建设，形成统一、开放、竞争、有序的文化市场体系。加强民族民间文化的保护与开发利用，进一步搞好文物保护工作。加强农村乡镇和城镇社区文化设施建设，完成广播电视数字化改造。加快图书馆、博物馆、文化馆、档案馆等公益性文化设施建设。

九、以解决人民群众最关心、最直接、最现实的问题为各项工作落脚点，努力建设和谐贵阳，增创社会建设和管理新优势

（一）千方百计扩大就业。强化政府促进就业的公共服务职能，深化户籍制度、税收制度、城镇就业管理制度等改革，健全就业服务体系，加快建立职业技能培训机制，推行劳动预备制度、职业资格证书制度，继续实施和完善鼓励企业增加就业岗位、加强就业培训的优惠政策，完善对困难群众的就业援助制度，建立促进扩大就业的有效机制。规范劳动力市场秩序，保护劳动者合法权益。累计增加城镇就业20万人以上，城镇登记失业率控制在4.5%以内。

（二）加快完善社会保障体系。建立健全与我市经济发展水平相适应的社会保障体系，完善城镇职工养老、医疗、失业、工伤和生育保险制度。推进机关事业单位养老保险制度改革，逐步将进城务工人员纳入城镇社会保障范围。进一步完善和规范农村最低生活保障制度。增加财政的社会保障投入，多渠道筹措社会保障基金，逐步做实个人帐户。加强社会福利事业建设，完善优抚保障机制和社会救助体系。支持社会慈善、社会捐赠、群众互助等社会扶助活动。完善社会保障信息网络建设，完善按劳分配为主体、多种分配方式并存的分配制度，坚持各种生产要素按贡献参与分配，通过加强管理和再分配手段，注重社会公平，调节过高收入，扩大中等收入者的比重，增加低收入者的收入。完善社会住房保障体系，逐步改善城镇低收入家庭住房困难状况。

（三）提高人民群众健康水平。加大政府对卫生事业的投入力度，完善突发公共卫生事件应急指挥体系、疾病预防控制体系、医疗救治体系、卫生执法监督体系和城乡医疗服务体系。加强计划免疫和重大传染病、地方病的综合防治工作，继续开展群众性爱国卫生运动。加强妇幼卫生保健，大力发展社区卫生服务。加快推进医疗体制改革，完善农村新型合作医疗制度，合理配置医疗卫生资源，整顿药品生产和流通秩序，逐步解决群众看病难、看病贵问题。贯彻计划生育基本国策，稳定人口低生育水平，积极推行优生优育，做好出生缺陷干预，提高出生人口素质，有效治理出生人口性别比偏高的问题，加强对流动人口的综合治理工作。实现计划生育依法管理、村（居）民自治，继续开展创建优质服务先进区（县）活动，实施农村计划生育奖励扶助制度和“少生快富”工程。加强城乡体育设施建设，大力开展全民健身运动，提高竞技体育水平。顺应人口老龄化的趋势，大力兴办服务于老年人的各项社会事业，逐步形成比较完善的老年服务体系。

（四）积极开展争创“全国文明城市”活动，深入推进“平安贵阳”建设。以争创“全国文明城市”为目标，深入贯彻落实《公民道德建设实施纲要》，重点抓住道德规范宣传教育、各类文明创建活动、建设生态文明、营造文明交通秩序、创造科学文化氛围五个重点，紧扣“道德教育、基础建设、活动载体、舆论氛围、评价考核”等五个环节，全面推进精神文明建设，促进我市经济发展和社会全面进步。切实加强社会治安综合治理和社会治安防控体系建设，强化禁毒工作，依法严厉打击各种违法犯罪，整顿和规范市场经济秩序，努力营造“居住在贵阳、创业在贵阳、平安在贵阳”的良好环境。完善信访工作责任制，建立健全社会预警体系和应急救援与社会动员机制，切实保障公共安全。高度重视安全生产，切实抓好煤矿、交通、消防安全监督管理，切实预防和有效减少安全生产事故。加强对各种自然灾害的预测预报和防灾减灾工作。

2006年的主要工作

2006年是我市“十一五”规划的开局之年，是保持经济增长良好势头、乘势前进的关键一年。根据中央、省经济工作会议和市委七届十五次全会的部署，综合分析当前的形势和工作要求，2006年全市经济和社会发展的主要预期目标是：生产总值增长13%。其中，第一产业增长8%，第二产业增长15%，第三产业增长13%；全市固定资产投资总额比上年净增50亿元；财政总收入和地方财政收入均增长13%，力争和全省保持相同增幅；社会消费品零售总额增长14%；进出口总额增长10%，利用外资增长15%；城市居民人均可支配收入实际增长10%，农民人均纯收入实际增长10%；城镇登记失业率控制在4.5%以内；人口自然增长率控制在6‰以内；万元生产总值综合能耗比上年下降5个百分点。全面完成市委、市政府为民办好的十件实事。

为实现上述目标任务，突出抓好以下十项工作：

一、坚定不移地实施“工业强市”战略，促进经济增长方式转变，在增加经济总量、提高经济效益上取得新进展

一是不断增强自主创新能力，培育壮大新兴产业和高新技术产业，重点发展电子信息产品、新材料、现代药业等高新技术产业。强力推进金阳科技产业园建设，基本完成东区基础设施建设，建成海关及保税仓储区并投入使用。拓展高新开发区规划面积，抓好区内标准厂房建设，建成投产12个以上产业化项目。加快国家贵阳片式电子元器件产业园、贵阳国际新材料产业化基地建设，重点推进微硬盘基地建设，扩大450兆赫兹CDMA手机和京瓷CDMA手机产量，快速推进GSM手机生产线建设，抓好汉方国家二类抗肿瘤注射新药、信邦针剂GMP改造、华城楼宇中央空调能源管理系统等高新技术产业项目。抓好中国西部（贵阳）高新技术产业研发生产基地、贵阳循环经济研究院等研发中心、企业技术中心的建设，建成贵阳微软技术中心、贵阳CA认证中心。组织实施国家、省和市重点技术创新项目50个。确保高新技术产业工业增加值增长25%。加大信息化建设力度，继续推进“数字贵阳”建设。着力推进贵阳地理信息系统一期工程建设，重点启动“金阳新区规划、建设和管理信息系统”建设和“1261”企业信息化工程建设。二是加快发展支柱产业和特色产业，促进工业提速增效。着力优化提升磷化工、铝及铝加工、煤化工、装备制造、卷烟、药业、能源、食品加工等八大优势产业，提高市场占有率和竞争力。加快贵阳卷烟厂易地搬迁、开磷集团120万吨磷铵、兖矿年产50万吨合成氨等一批重大项目的建设进度；启动清镇电厂异地搬迁，力争开工建设为清镇电厂搬迁配套年产75万吨的两个煤矿坑口；促进贵州铝厂四期电解铝、40万吨氧化铝扩建项目尽快达产增效，支持水晶集团发展煤化工产业。积极支持企业技术改造，重点抓好贵州轮胎厂年产200万条半钢子午线轮胎、兰花水泥二期等项目。积极支持制药企业做大做强，确保医药业工业增加值增长30%。全市工业增加值增长20%左右。

二、扎实推进社会主义新农村建设，进一步做好“三农”工作，在促进农业现代化、推动县域经济突破上取得新进展

一是坚持规划先行，在已完成村镇总体规划的基础上，完成100个新农村规划。二是加快农业和农村经济结构调整，着力提高农村经济发展水平。重点建设乌当、白云花卉产地批发市场，加快花卉市场二期建设，新增1000个以上花卉大棚，花卉产值力争突破1亿元，使花卉成为全市农业新的经济增长点；全力支持“老干妈”风味食品有限公司等辣椒制品和食品加工的发展，力争年产值达到20亿元；切实抓好养殖小区建设，力争新增养殖小区200个，新增奶牛1万头。重点建设公路沿线蔬菜产业带，大力发展外销蔬菜。新增10家以上市级龙头企业。三是加强农业农村基础设施建设，着力改善农村生态环境和农民生产生活条件。全市新增投资3亿元以上用于改善农村基础设施建设。利用亚行贷款，开工建设乌当渔洞峡等水库，完成鱼简河水利工程，继续实施万户小水池工程，解决农村3万人饮水安全。完成1000公里农村道路改造和500公里村寨串户路硬化，完成市级退耕还林5000亩。继续实施“四改一气”农村生态能源建设，新建沼气池7万口及配套项目，建设10个农村公共信息服务直通车试点。四是加大农村合作组织建设和农民培训、转移力度，不断提升农民组织化、市场化、专业化水平。完成农民实用技术培训20万人次，非农技能培训2.5万人，转移就业2万人。五是加大扶贫攻坚和财政支农力度，促进公平发展。

三、大力实施项目带动战略，在加大投资拉动力度、构筑产业竞争优势上取得新进展

一是积极谋划和组织项目建设，扎实做好项目前期工作。进一步搞好项目库建设，积极做好城市轻轨等一批大型项目的规划、论证的前期工作。对西郊水厂等历年财政性投资建成未转固的200多个项目进行清理，完成“投转固”工作并实现市场化运作。二是加快实施重大项目带动，保质保量抓好项目建设。开工建设新庄污水处理厂、小河污水处理厂二期、金竹污水处理厂、朱昌污水处理厂，完善清镇污水处理厂污水收集系统。开工建设贵惠路、西南环线、宅吉路、四方河联络线等项目，完成陈亮河、原贵阳三中地块、东出口环境综合整治。继续配合做好龙洞堡机场改扩建、贵阳绕城高速公路、贵遵高速公路扎南段、绕城铁路等项目建设。启动并建设贵阳到金阳的第三条便捷道路和金阳至清镇、开阳至久长高等级公路、贵阳公路主枢纽客货运输站场和区（市、县）客货车站建设，启动实施延安西路客车站二期站务大楼改造工程，确保完成贵开路、枣山路客运站站务大楼、第一服务车站站务大楼改建工程和10个乡（镇）客车站等项目；继续抓紧抓好贵阳客流园区枢纽总站拆迁和一期工程建设。进一步巩固与国开行等金融机构的合作关系，启动新一轮银政合作，力争在国内金融部门融资50亿元左右。按照世行贷款支持方向，抓紧做好贵阳公路枢纽管理信息指挥中心、倒岩路集装箱中转站、中曹司货运站等项目前期准备工作，争取列入世行贷款项目并早日开工。

四、大力发展服务业，在增加城乡人民收入、扩大就业和促进消费上取得新进展

一是以发展现代服务业为重点，促进服务业全面发展。重点启动扎佐物流园区建设。加大对青岩古镇、红枫湖景区、乌江峡风景区、贵阳森林野生动物园等景点的保护和开发建设力度，努力打造并形成一批精品旅游线路。启动贵阳城市经济圈建设。以遵崇路开通为契机，吸引更多的川渝地区人士到贵阳旅游投资。大力发展会展经济，精心组织兰花博览会、“黔菜”主题美食节、风筝节、红枫湖旅游节、花溪之夏艺术节等展会，配合省举办好“黄果树瀑布节”。二是完善扩大消费政策措施，大力培育新的消费热点。积极引导和促进居民扩大住房、家用轿车、电信、旅游、文化、教育、体育、休闲娱乐和其他服务性消费。三是加快市场建设。着力抓好12个重点市场的建设、培育工作，继续抓好浙江商城、金阳商贸走廊及将富水路改造成为商业步行街的规划和建设，加大城中心区农贸市场的建设管理力度，云岩区、南明区、小河区各完成一个“三绿”试点示范市场建设，逐步消除马路市场和占道经营。

五、加强城市规划、建设和管理，在完善城市设施、提升城市品位上取得新进展

一是加强城市规划和管理。启动我市城市总体规划和重点区域控制性详细规划的修编工作。切实抓好南明河景观改造、街道亮丽、城区美化、绿化及新建公厕、垃圾台等工程。按照创建全国文明城市的总体要求，以“创建文明城市、建设和谐贵阳”为目标，继续深入开展“为民办实事、满意到万家”活动，深入推进城市综合执法，加大城市交通秩序、环境卫生、噪声管制、垃圾无害化处理和户外广告等的规范管理工作，加强对违法建筑、占道经营、乱停乱放等的查处力度，加大对两城区夜市摊点的规范化管理，逐步实现城市环境卫生长效管理。做好城市天际绿线保护工作，对城区25度以上山体生态资源实施严格保护，凡破坏城市山头绿地进行房地产开发的项目一律不予审批。实施红枫湖环湖林带和环湖旅游道路的建设。二是加大旧城改造力度。实施贵惠路、四方河联络线等道路改造工程，对中心区道路进行改造，并对城区部分干道进行沥青道路改造，修建2个以上人行过街系统。确保全市经济适用住房开工120万平方米，竣工80万平方米。抓好东出口和中心环北线的环境综合整治工作；大力实施“城中村”改造，重点在云岩、南明、小河、白云各启动一片城中村改造项目，进一步加大拆除违章建筑力度，有效遏制住乱搭乱建势头。三是掀起金阳新区建设新高潮。落实好金阳新区鼓励投资扶持政策，切实抓好政务服务中心建设。完成市级行政中心二期搬迁工作，建设市政公共设施、科技园区、体育文化等八大城市功能板块，抓好体育中心、科技中心、工人文化宫、青少年活动中心、妇女儿童活动中心、广电中心、市消防支队、市武警支队、市交警指挥中心、市直机关职工经济适用住房等工程的建设。全力配合、支持贵州大学、省商业专科学校、省电大、省检察院和一批总部、大公司搬迁工程，完成贵阳一中二期、贵阳三中新校和金阳医院建设。加快推进金阳新区40平方公里的基础设施建设。四是加大小城镇建设力度。切实抓好以区（市、县）、乡（镇）政府所在地为重点区域的城镇总体规划及土地利用总体规划的修编工作和新农村规划。大力完善城镇基础设施建设力度，严格规范农房规划、审批和建设。

六、进一步深化改革、扩大开放，在完善市场经济体制、招商引资工作上取得新进展

一是进一步深化国有企业改革。向金融部门争取部分贷款用于支持国有企业改革改制，力争国有商贸流通企业全部从一般性竞争行业中退出，国有工业企业改革重组取得重大突破。基本完成商控、工控、建控公司所属企业的改制工作。二是搞好花溪区、修文县农村综合改革试点工作，基本完成乡镇机构改革。三是深化投融资、财税和金融体制改革。加大贵阳市商业银行改革力度，支持其做强做大。加快推进农村信用社改革和发展。四是大力发展非公有制经济。抓紧出台《关于大力发展非公有制经济的决定》，力争非公有制经济在全市经济总量中所占比重上升2个百分点。五是扩大对外开放，努力提高招商引资的规模和质量。精心策划、组织好赴境外的招商推介会和长三角、珠三角、海峡西岸经济区招商活动。确保招商引资增长20%。

七、大力发展循环经济，在建设资源节约型和环境友好型城市、实现可持续发展上取得新进展

一是大力发展循环经济。全面启动全国循环经济试点城市建设工作，抓紧制定《加快循环经济发展的决定》，完成第一批循环经济试点项目，做好第二批试点项目的申报工作。启动实施欧盟《亚洲生态计划》在贵阳的项目，继续做好中德循环经济灯塔项目及中日环境合作示范城市项目，全面推进开阳磷煤化工循环经济示范基地、清镇铝、煤化工循环经济生态工业示范基地、息烽磷化工循环经济示范基地的建设工作。二是积极开展节能降耗和清洁生产工作。重点抓好贵化合成氨节能降耗国债节能改造项目，抓好清镇市全国节水试点城市工作，推进白云区污水再生利用。积极推进墙体材料革新和建筑节能工作，取消粘土实心砖的生产和使用，新建建筑必须执行节能率50%的设计标准，建筑围护结构达到隔热保温的规定。三是切实加大环境污染治理力度。加大红枫湖、百花湖等饮用水源点环境保护力度，对沿湖污染排放不合格企业一律关停，严格死水线保护制度，确保城市饮水卫生和饮水安全。加快危险品及固体废弃物处置中心、城市垃圾填埋场、污水处理厂等项目的建设，配合做好水晶集团汞污染治理项目前期工作，继续推进贵阳市水污染治理工程项目。

八、大力实施科教兴市、人才强市战略，在繁荣社会事业、促进经济社会协调发展上取得新进展

一是大力推进科技进步。切实抓好县级生产力促进中心建设，对贵阳风险投资公司的职能、规模进行提升，建立和完善高新技术风险投资机制，建设贵阳循环经济研究院和天然植物药业研发中心，启动中国西部（贵阳）高新技术产业研发生产基地建设。二是优先发展教育事业，加快人才队伍建设。切实抓好“两免一补”政策的落实，创建21所标准化中小学、改造4所城区初中薄弱学校，改扩建8所公办学校，扩大外来务工子女接受公办义务教育的数量。建成贵阳三中新校，抓好贵阳一中二期、二中、六中、民中改扩建工程，做好实验三中、民中、息烽中学、修文中学四所学校申报省级示范性高中的工作。加快建设贵阳学院和贵阳护理职业学院，组建贵阳职业技术学院和贵阳学院教师教育分院。三是大力发展文化和体育事业。抓紧出台《关于加快发展文化产业的决定》，抓好贵阳大剧院管理使用和县乡文化馆、社区文化活动中心阵地建设，启动金阳体育中心的建设工作，精心举办好贵阳市第十届运动会。四是大力发展卫生事业。全面实施新型农村合作医疗制度，完成贵阳市传染病医院改扩建工程并投入使用，建成金阳医院并投入使用，改造9个乡（镇）卫生院，建设村卫生室100个；做好艾滋病、结核病等重大疾病和人畜（禽）共患疾病防控工作。加强农村环境卫生建设，创建文明卫生村寨19个。五是加强人口与计划生育工作。确保人口自然增长率控制在6‰以内，出生率控制在11.5‰以内，符合政策生育率达94%以上。

九、着力解决关系人民群众切身利益的突出问题，在维护社会稳定、构建和谐贵阳上取得新进展

一是努力扩大就业和再就业。确保新增就业岗位3.8万个，城乡统筹就业6.4万人，城乡统筹培训4.4万人。二是加强社会保障体系建设。推进养老、医疗、失业、工伤、生育等社会保障制度改革，提高城镇居民最低生活保障标准，城镇登记失业率控制在4.5%以内。三是加强安全生产工作。进一步完善安全生产指标控制体系，落实安全工作责任制，确保安全生产重特大事故发生率和死亡率控制在省下达指标范围内。四是加强禁毒工作。积极开展无毒社区、单位和无毒区（市、县）创建活动，将禁毒工作纳入政府目标考核，实行“一票否决”。四是切实维护社会稳定，继续深入开展“严打”和各种专项斗争，全力推进“平安贵阳”创建活动。建立健全公共突发事件应急处理机制，抓好构皮滩库区移民安置等涉及面广的维稳工作，积极预防和处置群体性事件，切实维护社会稳定。

十、深化行政体制改革，切实转变政府职能，在建设求实、务实、高效、廉洁型政府上取得新进展

一是深化行政体制改革，全面正确履行政府职能。按照精简、统一、效能的原则和决策、执行、监督相协调的要求，深化行政管理体制改革和行政审批制度改革，实现政企分开、政事分开，切实把政府职能转到经济调节、市场监管、社会管理和公共服务上来。切实提升、完善政务服务中心功能，发展电子政务，努力降低行政成本，提高行政效率。二是强化依法行政，全面推进科学民主决策。进一步贯彻落实《国务院依法行政实施纲要》，坚持依法治市，加大责任追究力度，全面落实政府行政执法责任制和过错追究制，做到依法决策、依法行政。各级政府要自觉接受同级人大及其常委会的法律监督和工作监督，自觉接受同级政协的民主监督，认真听取民主党派、工商联、无党派人士和各人民团体的意见，接受新闻舆论和社会公众监督，重视人民群众对政府机关及其工作人员的监督，支持监察、审计部门依法独立履行监督职责。进一步健全科学民主决策机制，建立和完善重大问题集体决策制度、专家咨询制度、社会听证制度和决策责任制度。三是大力弘扬求真务实的作风，狠抓工作落实。强化目标责任管理机制，切实做到目标任务、重点要求、关键措施、时间进度、责任人员“五明确”，不断强化目标管理的导向作用。结合《公务员法》的实施，健全用人导向机制，强化公务员绩效管理。强化督促检查机制和考核奖惩机制，对重大决策部署、重点工作、重大项目、为民办实事项目进展情况加强跟踪督查，及时兑现奖惩。四是切实加强党风廉政建设。全面贯彻落实中央《建立健全教育、制度、监督并重的惩治和预防腐败体系实施纲要》，加强理想信念和廉洁从政教育，完善领导干部廉洁自律制度，落实党风廉政建设责任制，从源头上遏制腐败现象的滋生。

各位代表！“十五”伟业送走了辉煌的过去，“十一五”宏图迎来了壮丽的前景，让我们紧密团结在以胡锦涛同志为总书记的党中央周围，高举邓小平理论和“三个代表”重要思想伟大旗帜，按照胡锦涛总书记对贵阳市“做表率、走前列”的要求，认真贯彻、落实党的十六届五中全会、省委九届八次全会及市委七届十五次全会精神，立足新起点，增创新优势，实现新跨越，坚定信心，振奋精神，开拓进取，扎实工作，为全面完成国民经济和社会发展第十一个五年规划，实现贵阳市经济社会发展的历史性跨越而努力奋斗。
